# Supplementary material for: A modern automated patch-clamp approach for high throughput electrophysiology recordings in native cardiomyocytes
Source: Commun Biol. 2022 Sep 15;5:969. doi: 10.1038/s42003-022-03871-2 (PMC9477872; doi:10.1038/s42003-022-03871-2)
Supplement: Supplementary file 2 — Supplementary Information [file 42003_2022_3871_MOESM2_ESM.pdf]

A modern automated patch-clamp approach for high throughput electrophysiology recordings in native cardiomyocytes

Supplementary Information

Fitzwilliam Seibertz[1,2,3], Markus Rapedius[4*], Funsho E. Fakuade[1,2,3], Philipp Tomsits[5,6,7], Aiste Liutkute[1,2,3],Lukas Cyganek[2,3,8], Nadine Becker[4], Rupamanjari Majumder[2,9], Sebastian Clauß[5,6,7], Niels Fertig[4*], Niels Voigt[1,2,3*]

[1]Institute of Pharmacology and Toxicology, University Medical Center Göttingen, Georg-August University Göttingen, Germany

[2]DZHK (German Center for Cardiovascular Research), partner site Göttingen, Germany

[3]Cluster of Excellence "Multiscale Bioimaging: from Molecular Machines to Networks of Excitable Cells" (MBExC), Georg-August University Göttingen, Germany

[4]Nanion Technologies GmbH, Munich, Germany

[5]Department of Medicine I, University Hospital, LMU Munich, Munich, Germany

[6]Institute of Surgical Research at the Walter-Brendel-Centre of Experimental Medicine, University Hospital, LMU Munich, Munich, Germany

[7]DZHK (German Centre for Cardiovascular Research), Partner Site Munich, Munich Heart Alliance (MHA), Munich, Germany

[8]Stem Cell Unit, Clinic for Cardiology and Pneumology, University Medical Center Göttingen, Georg-August University Göttingen, Germany

[9]Biomedical Physics Group, Max Planck Institute for Dynamics and Self Organisation, Gottingen, Germany

***Corresponding Authors:**

Markus Rapedius, Nanion Technologies GmbH, Ganghoferstraße 70a,

80339 Munich, Germany; E-mail: markus.rapedius@nanion.de

Niels Fertig, Nanion Technologies GmbH, Ganghoferstraße 70a, 80339

Munich, Germany; E-mail: niels.fertig@nanion.de

Niels Voigt, Institute of Pharmacology and Toxicology, University

Medical Center Göttingen, Georg-August University Göttingen, Robert-

Koch-Straße 40, 37075 Göttingen, Germany; E-mail:

niels.voigt@med.uni-goettingen.de

Table of Contents

Supplementary Figures **3**

Supplementary Figures

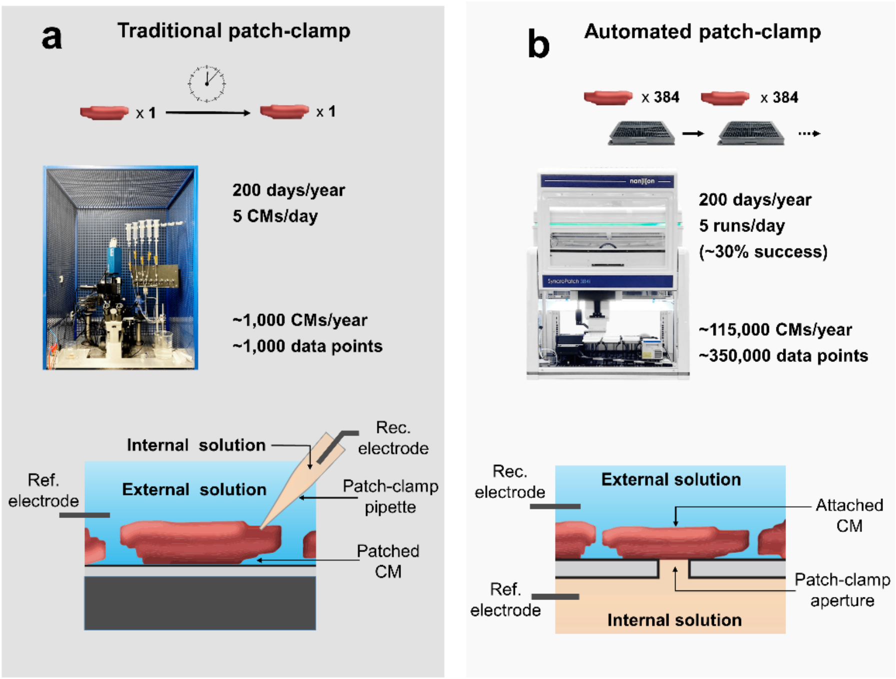

Supplementary Figure 1. Method comparison: traditional patch-clamp vs automated patch-clamp (APC) for investigation of native mammalian cardiomyocytes (CMs). a, Traditional patch-clamp requirements in which only one cell can be laboriously measured at a time by a skilled operator. **b,** APC summary detailing measurement capabilities of upwards of 600 CMs per day and an annual data point increase of 35,000%. For the purposes of this figure: ‘Data points’ are defined as currents/action potentials measured per cell e.g $I_{Ca,L}$, AP, and I_{K1} (3 parameters) as seen in the CAPER protocol. They do not include the numerous recordings possible using pharmacological screening of a single current/AP.

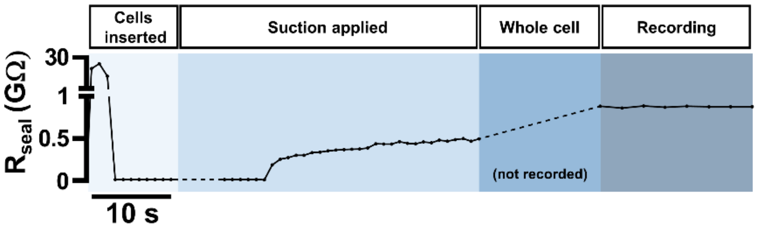

Supplementary Figure 2. Representative time course of seal resistance (R_{Seal}) during cell addition, suction application, whole cell configuration and eventual experimentation. Before whole cell configuration is achieved, transiently elevated external Ca^{2+} levels (5 mM max) help to foster giga seal formation.

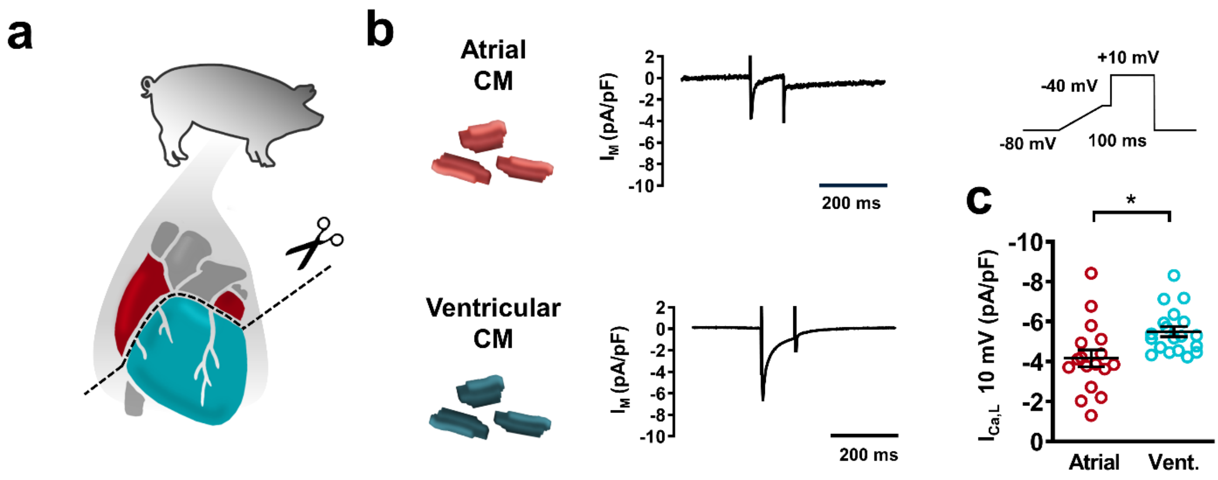

Supplementary Figure 3. Manual patch-clamp of native cardiomyocytes (CM) from the same swine construct as used in this study. a, Schematic of swine cardiac tissue harvesting. **b**, Representative L-type calcium current ($I_{Ca,L}$) recorded from atrial CM (upper) and ventricular (vent.; lower) CM. Far right: voltage protocol for $I_{Ca,L}$ acquisition. **c,** $I_{Ca,L}$ current density measured at +10 mV (n = 17 atrial vs 19 ventricular from 3 animals). Data are mean±SEM. *$P<0.05$ vs ventricular.

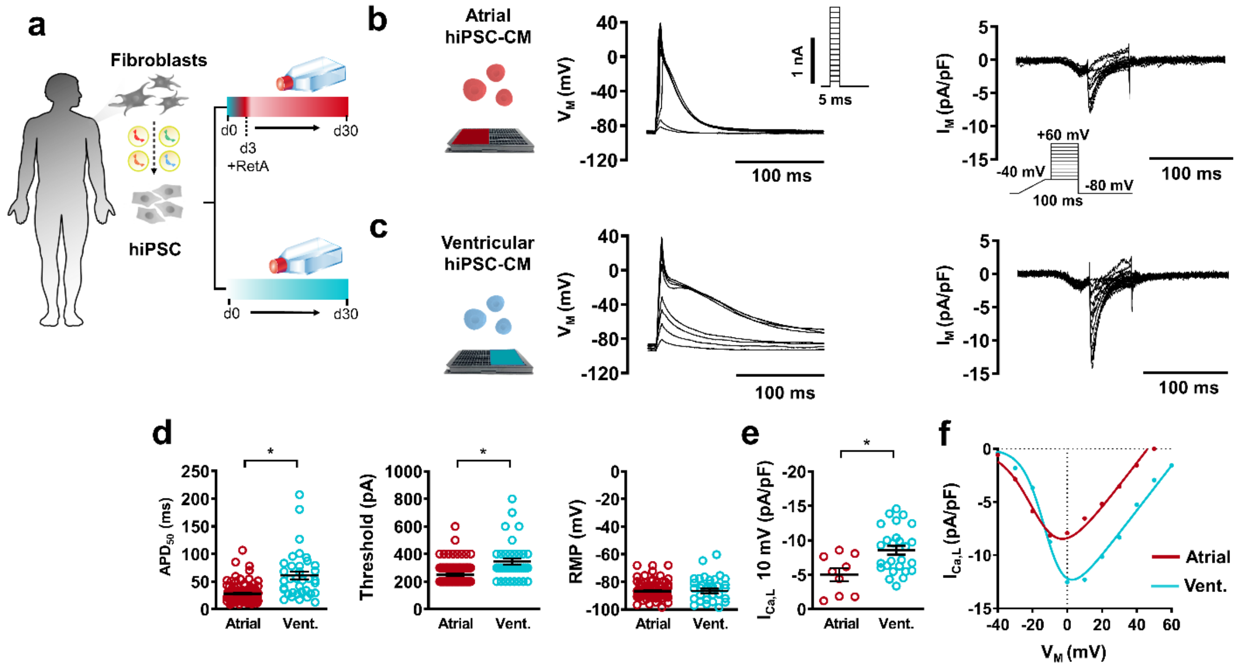

Supplementary Figure 4. Automated patch-clamp (APC) of human atrial and ventricular induced pluripotent stem cell-derived cardiomyocytes (hiPSC-CM). **a,** Schematic of human dermal fibroblast harvesting, reprogramming into induced pluripotent stem cells (hiPSC) and subsequent differentiation into ventricular (Vent.) and atrial cardiomyocytes with the latter receiving retinoic acid (RetA) early in differentiation for atrial lineage confirmation. **b**, Representative action potential (AP; left) and L-type calcium current ($I_{Ca,L}$; right) recorded from separate atrial hiPSC-CMs. insets: current protocol for AP acquisition and voltage protocol for $I_{Ca,L}$ acquisition. **c**, Representative AP (left) and $I_{Ca,L}$ (right) recorded from ventricular hiPSC-CMs. **d**, AP duration at 50% repolarization (APD_{50}; left), current threshold which first elicited an AP response (center) and resting membrane potential (RMP; right; n = 108 atrial vs 35 ventricular). **e**, $I_{Ca,L}$ current density measured at +10 mV (n = 9 atrial vs 26 ventricular; right). **f**, A single representative current-voltage (I-V) relationship plot for $I_{Ca,L}$ in an individual atrial and ventricular hiPSC-CM. Data are mean±SEM. *P<0.05 vs ventricular.

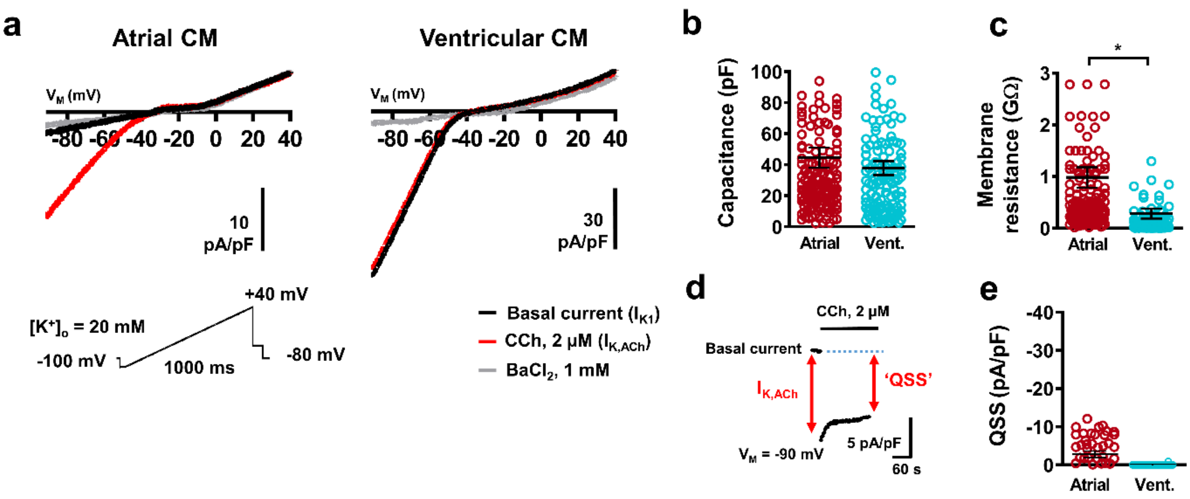

Supplementary Figure 5. Features of inward rectifier currents measured in native atrial and ventricular cardiomyocytes (CM) using automated patch-clamp. a, Representative uncorrected basal inward rectifier current (I_{K1}) in atrial (left) and ventricular (vent.; right) CMs with superimposed uncorrected peak (initial) acetylcholine-activated inward rectifier ($I_{K,ACh}$) current following carbachol (CCh) application, and during full block with $BaCl_2$ application, all during a depolarizing ramp voltage protocol (lower). **b**, Cell capacitance. **c**, Membrane resistance, calculated through dividing the driving force of K^+ (30 mV) by the absolute Ba^{2+} sensitive current at -90 mV. **d**, Detailed representative timecourse of CCh application and the eventual desensitization to a quasi steady-state (QSS). **e**, QSS current. Data are mean±SEM. **P*<0.05 vs ventricular. n=number of atrial (151) and ventricular (143) CMs from 3 animals (**b, c, e**).

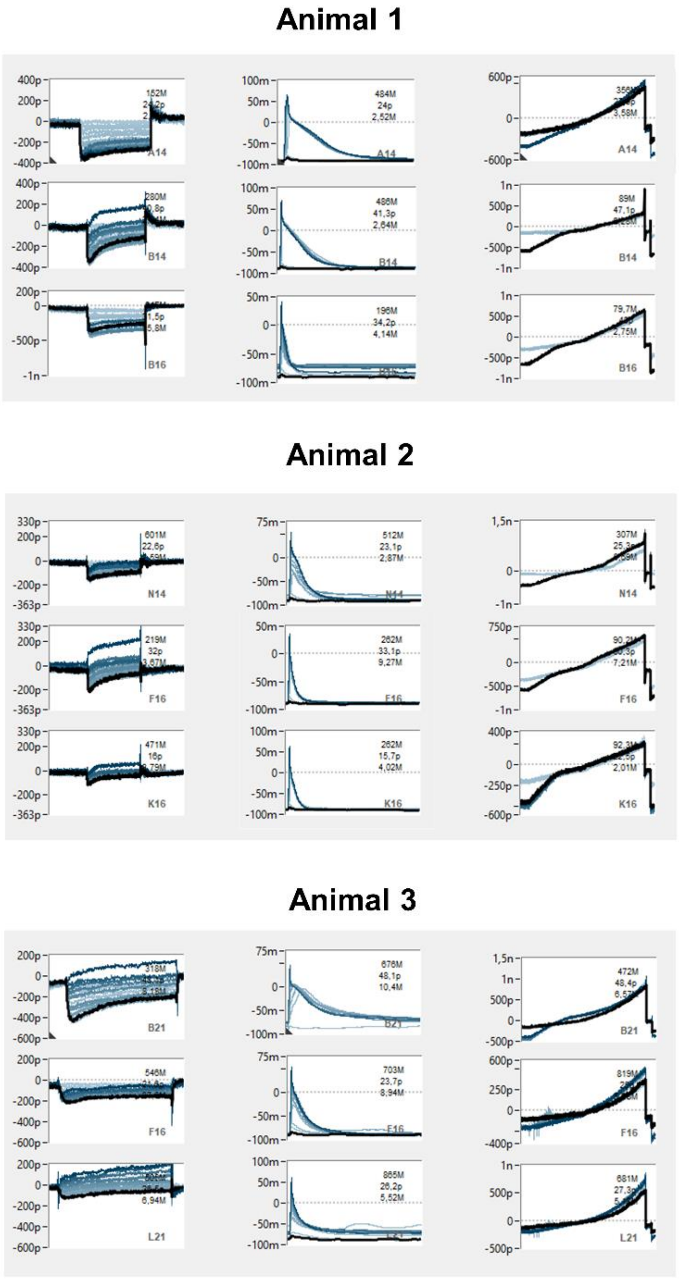

Supplementary Figure 6. Representative raw data of atrial cardiomyocytes undergoing the multi-current Calcium, Action Potential and inward rectifiER (CAPER) protocol. Direct screenshots from the Nanion DataControl 384 software during recording analysis. One row indicates one cell. A single animal was measured per day. Three representative cells are shown for each animal. The Y axis of each recording indicates the membrane current expressed as picoamperes (p) or nanoamperes (n) in the screenshots of $I_{Ca,L}$ (left side) and I_{K1} (right side), or membrane voltage expressed as millivolts (m; center).
