# Supplementary material for: A modern automated patch-clamp approach for high throughput electrophysiology recordings in native cardiomyocytes
Source: Commun Biol. 2022 Sep 15;5:969. doi: 10.1038/s42003-022-03871-2 (PMC9477872; doi:10.1038/s42003-022-03871-2)
Supplement: Supplementary file 3 — Description of Additional Supplementary Data [file 42003_2022_3871_MOESM3_ESM.pdf]

Description of Additional Supplementary Files

File name: Supplementary Video 1.

Description: Multi-current Calcium, Action Potential and inward rectifiER (CAPER) results from atrial cardiomyocytes in three dimensions.

File Name: Supplementary Data 1

Description: Source data behind graphs in the paper
